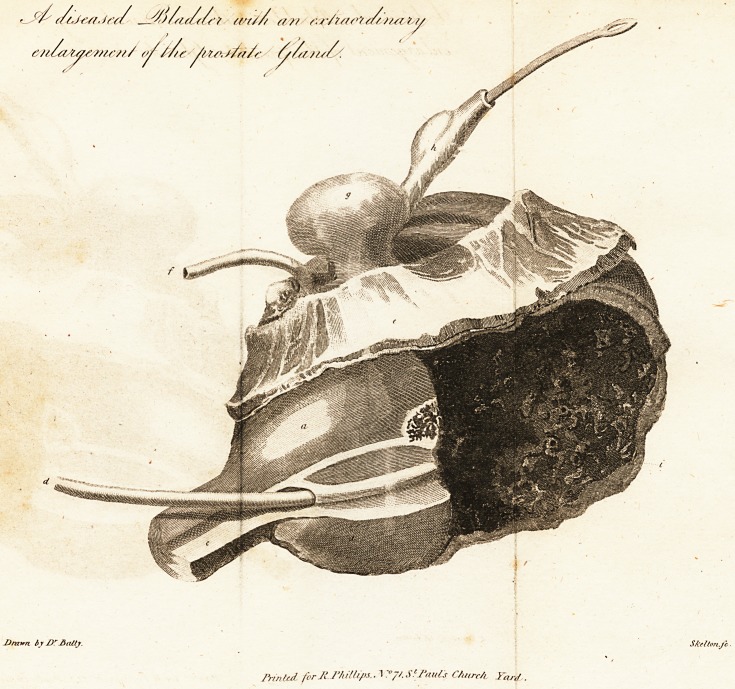# Case of a Diseased Bladder, with Enlargement of the Prostate Gland, and Other Morbid Appearances

**Published:** 1800-11

**Authors:** Edward Ford

**Affiliations:** Surgeon to the Westminster General Dispensary


### *Case of a diseased Bladder, with Enlargement of the Prostate Gland, and other morbid Appearances.* By EDWARD FORD, F. S. A. Surgeon to the Weſtminſter General Diſpenſary.

[ With an Engraving. ]

To the EDITORS of the MEDICAL and PHYSICAL JOURNAL.

Gentlemen,

AS the Drawing which accompanies this caſe, exhibits ſome uncommon appearances, I have ſent it to you for publication in the Medical and Phyſical Journal, if you think it will not be unacceptable to your readers. Specimens of ſacculi in the bladder, and of falſe paſſages in the urethra, are probably to be ſeen amongſt the morbid preparations of many anatomiſts; but the hiſtories of ſuch diſeaſes have not been ſo frequently offered to the public as their importance requires.

Bonetus has recorded inſtances of ſtones depoſited in ſacs of the bladder; and Morgagni has enriched this ſubject by the recital of caſes, in which a ſtone was ſometimes diſtinctly felt, and at other times could not be perceived, the cauſe of ſuch uncertainty being afterwards found to ariſe from ſacculi; ſo that this illuſtrious writer thought it neceſſary to warn the lithotomiſt, mift, as he values his own reputation, or the fafety of his patient, not to operate, unlefs he diftinctly feels a ftone in the cavity of the bladder at the time of operating.

The fubject of this paper was feventy-four years of age, and had been for feveral years liable to complaints in the urinary paffage, for the relief of which, he had ufed fuch means as had been at various times prefcribed for him; he had occafionally fuffered under fits of the gout, but the general ftate of his health might otherwife be faid to be good: the earlier part of his life was fpent in the Eaft Indies, but by temperance and regularity he had preferved his conftitution tolerably free from the difeafes incident to hot climates.

I was called upon to fee him about a year before he died; he was then diftreffed exceedingly by a conftant and almoft perpetual irritation to void his urine, attended with confiderable pain, and an itching fenfation in the urethra, almoft intolerable; a copious difcharge of mucus, mixed with blood, generally followed his urine; and whenever this happened, his pain was confiderably aggravated; he fometimes had a fuppreffion of his urine, which was eafily relieved by the ufe of the flexible catheter; at other times it paffed involuntarily, and particularly if any degree of force had been ufed in the introduction of a bougie or catheter.

Thefe difficulties were increafed by a conftipation of the bowels, a fymptom which was eafily explained; for, by examining with the finger introduced into the rectum, it was found that the proftate gland was much enlarged, and protruded backwards into the inteftine, fo that it neceffarily obftructed the paffage of the fæces. The urethra was examined by a middle fized bougie, and found to be free from ftricture, or any other embarrafsment but that produced by the enlargement of the proftate gland, a circumftance which impeded, in fome meafure, the paffing of the bougie into the neck of the bladder.

The fymptoms under which he laboured, evidently requiring a further inveftigation by a found or filver catheter, in order to afcertain whether or not there was a ftone in the bladder, this trial was made by feveral furgeons; but it was found impracticable to introduce either of thefe inftruments; the filver catheter would apparently pafs up the whole length of the urethra, but upon attempting to give it that kind of turn neceffary for this examination, it was found that its point was in a confined ftate, that it could not be liberated from fome ftricture it underwent; in fhort, that it had not paffed fairly into the cavity of the bladder. From the frequent returns of exceffive pain, the patient was often folicitous of having his difeafe examined as accurately as poffible; and I was once fortunate

 enough enough to introduce a ſound into the bladder, but was not able to diſcover that it contained a calculus. He proceeded in this melancholy way for ſeveral months, with ſhort intervals of eaſe procured at times by the drawing off his urine, by anodyne gliſters, by aperient medicines, by the warm bath, and ſuch other temporary expedients as the exigence of the moment ſeemed to require.

In the beginning of laſt Auguſt his health gradually declined; his appetite failed; the bowels became more conſtipated; the deſire of voiding his urine became more frequent and painful; he was tormented with a violent pain in his back and loins; and the diſcharge from his bladder was bloody, purulent, and fœtid; ſoon after, a tenſion took place in the cavity of the abdomen, ſucceeded by a colliquative purging, and he became heavy, comatoſe, and died.

The body was opened on the ſucceeding day, in the preſence of Mr. Pooly, his apothecary; and the appearances on diſſection fully explained the ſymptoms which the patient had ſuffered.

The thoracic viſcera were in a ſound and healthy ſtate.

In the cavity of the abdomen, the liver, gall bladder, omentum, ſpleen, ſtomach, and pancreas ſhewed no marks of diſeaſe; ſome portions of the inteſtinal canal were a little inflamed, the kidneys were both conſiderably ſo; the bladder was contracted, and contained a ſmall quantity of purulent urine; and a ſtone about the ſize of a cheſnut was found ſlightly adhering to an ulcerated ſurface near the proſtate gland; the internal membrane of the bladder was in a black, gangrenous ſtate, very offenſive, and nearly approaching to mortification.

On the left ſide of the bladder, near the termination of the ureter, was a pouch communicating with the bladder, and correſponding in ſize and figure with the ſtone, which poſſibly might have been formed in that receſs; and afterwards paſſing from thence, fixed itſelf to the neck of the bladder, thereby increaſing the painful ſymptoms of this diſeaſe.

In the lower part of the left ureter there was another ſmall ſac, which alſo contained a calculus.

The proſtate gland was enlarged very conſiderably.

On examining the urethra, a ſmall valvular orifice was ſeen on its ſuperior part, about an inch from the neck of the bladder, to which the opening immediately led, without penetrating its inner coat; it was ſufficiently large to admit the bulbous end of a catheter; and from its ſituation, it is highly probable that whenever a ſilver catheter was introduced for the purpoſe of drawing off the urine, it overcame the ſlight reſiſtance of this valve, and paſſed into the falſe paſſage; whilſt, on the contrary, an inſtrument of a ſofter texture, a bougie, or catheter made of of elaſtic gum, would glide eaſily into the bladder. It is impoſſible to ſay at what period this preternatural opening was formed; whether it was made by the paſſing of bougies in an early part of life, or whether it was formed at a later period, when the proſtate gland increaſing in bulk, rendered the introduction of the ſilver catheter an operation of difficulty.

The inflammation in the kidnies, the ſtone in the bladder, the ulceration which it had ſeemed to produce, and the conſequent gangrenous ſtate of the parts, manifeſtly explain every circumſtance which attended the final termination of this caſe; whilſt the ſac, connected with the bladder, evidently ſhews the hazard of lithotomy, when the ſtone is not felt at the moment of operation, confirming the practical obſervation of the celebrated Morgagni.

*Golden-Square, Oct. 27, 1800.*

### Explanation of the Plate.

*a*, The proſtate gland enlarged.

*b*, Ulceration near the neck of the bladder, on which a ſtone was found ſlightly adhering.

*c*, The urethra.

*d*, A bougie paſſed into the preternatural opening in the urethra.

*e*, A portion of the rectum.

*f*, The vas deferens.

*g*, A ſac formed from the bladder.

*h*, The left ureter, with a ſac, which contained a ſmall calculus.

*i*, The internal coat of the bladder, in a ſtate nearly gangrenous.

## Figures and Tables

**Figure f1:**